# Youth health volunteers shaping the future of community-based digital health

**DOI:** 10.1371/journal.pdig.0001605

**Published:** 2026-07-21

**Authors:** Afzal Hussain, Rosnah Sutan

**Affiliations:** 1 Department of Public Health Medicine, Faculty of Medicine, National University of Malaysia, Jalan Yaacob Latif, Bandar Tun Razak, Cheras, Kuala Lumpur, Malaysia; 2 School of Biosciences Engineering and Technology, VIT Bhopal University, Kothrikalan, Sehore, Madhya Pradesh, India; Jos University Teaching Hospital, NIGERIA

## Abstract

The use of digital health technologies is advancing the provision of community health services, especially in low- and middle-income countries. However, limited attention has been given to the role of youth health volunteers in digital health systems. This article underscores the growing role of youth health volunteers as intermediaries, who are increasingly moving beyond traditional health promotion roles to include digital data collection, reporting and interpreting health information at the community level. While their roles have evolved, there are challenges in low digital literacy, ethical considerations on data privacy and informed consent, and accountability in using artificial intelligence (AI)-powered tools. The frequently held view that young people are “digital natives” who can readily navigate digital technologies neglects the need for training, capacity-building, and institutional support. This paper suggests capacity building, governance and the formalization of youth health volunteers are critical to building a digital health system that is equitable, trustworthy and responsive to community needs.

## Introduction

The rise of digital technologies is bringing about rapid changes to community health systems; yet little attention is given to the human actors who enable these changes. Among these are youth health volunteers, a vital yet often overlooked group of community engagement and health delivery, whose roles have not received attention in the face of digital health systems. While digital health technologies are increasingly being integrated into health systems, little conceptual and empirical insight has been given to the roles, challenges and governance considerations of youth health volunteers in these systems [1]. Here, youth health volunteers are defined as those who are typically between the ages of 15 and 35 years who are engaged in community-based health activities with varying degrees of formal or informal training and may be part of governmental, non-governmental and community-based health initiatives [2]. This paper specifically refers to youth health volunteers, rather than community health workers who are generally paid, well-trained, and formally embedded in health systems. By contrast, youth health volunteers tend to be engaged in a semi-formal or informal manner, with different levels of training and recognition [3]. Our paper takes an international outlook, with an emphasis on low- and middle-income countries (LMICs), where community-based health systems and digital technologies are changing rapidly.

Millions of people are involved in community health worker programmes worldwide, with estimates that more than 3.8 million community health workers are delivering critical health care, especially in low- and middle-income countries. A significant proportion of this workforce includes young and volunteer-based cadres who are increasingly participating in digital health initiatives through mobile health reporting, teleconsultation support and data-driven community engagement. The integration of digital health technologies in primary health services in many countries is now common, reflecting increased uptake and scale. These developments highlight the growing role of youth health volunteers in the digital health landscape, and the importance of support and training [4].

Digital technologies are increasingly influencing the way community health services are organized and delivered, bringing youth health volunteers to the forefront of implementing digital health solutions by virtue of their often-overlooked contributions. Caught between the ideals of community and health care provision, youth health volunteers are seeing their role extended as digital technologies become more integrated into health activities. From their existing role within health promotion and education, their brief has come to include the role of data gathering, reporting, and digital counseling.

In our opinion, youth health volunteers should now be regarded as more than just auxiliary workers but should be acknowledged for their roles as critical digital intermediaries, with their efforts formally included in national digital health strategies. As their role in data management, digital communication, and interpreting health information at the community level continues to grow, youth health volunteers are increasingly becoming key actors in achieving equitable implementation of digital health in low- and middle-income countries.

## Current landscape of digital health and youth volunteers

Mobile health apps, cloud reporting systems, and tele-consultation systems have also been widely employed by community health workers and youth volunteers in relation to maternal health care, adolescent health care, control of infectious diseases, and control of non-communicable diseases. Other recent studies and trends in the field of digital health have highlighted the use of machine learning algorithms that support decision-making tools to enable community health workers in risk stratification, patient triage, and prioritized patient follow-ups in resource-deficient settings where there is a lack of skilled professionals. In several low- and middle-income settings, community health worker programs have employed AI-enabled mobile apps to use patient-submitted data to identify people at risk. Predictive analytics have also informed outreach and vaccination programs. Recent implementation studies have shown improved efficiency of health service delivery and case detection using AI-enabled tools in community health.

For instance, in Malaysia, digital health programs such as the MySejahtera app, launched by the Malaysian Government, have been fundamental in supporting COVID-19 surveillance, contact tracing and health promotion, with community engagement supported by volunteers and health actors. Moreover, community health initiatives have also used mobile health technologies to support non-communicable disease surveillance and health promotion activities. Accredited Social Health Activists (ASHAs) in India have been involved in the use of mobile apps for community-level monitoring of maternal and child health, vaccination follow-up and reporting of health data. In Bangladesh, community health workers have incorporated digital platforms to enable teleconsultations and track infectious disease outbreaks. Similarly, in sub-Saharan African nations like Kenya and Uganda, digital health technologies have been introduced to support community health programs in real-time data collection, monitoring of patients and decision-making. Taken together, these cases provide evidence that youth and community health volunteers are no longer restricted to conventional health promotion activities and are now becoming increasingly involved in digital data collection, digital health communication, technology-assisted service delivery, and community engagement. Such changing responsibilities point to the growing importance of youth health volunteers as key intermediaries in digital health systems. These emerging roles serve to reinforce our primary argument that youth health volunteers must be acknowledged, appropriately trained, and institutionally supported as essential participants in equitable digital health systems [5–7].

These technologies have increased the efficiency and effectiveness of the community health care provision process where youth health volunteers have become crucial facilitators using technology to analyze and disseminate health information in the community. As digital brokers, here defined as the people who translate and communicate digital health information between health systems and communities, their role is crucial in health systems where community actors play a key role to ensure health services extension [8].

## Challenges in digital health implementation

However, the incorporation of modern digital resources within voluntary health activities mediated by volunteers poses many societal and structural concerns. Digitization tends to raise the bar within the health sector about the expectations for health data as well as responsiveness to digital services. It also places additional responsibilities and expectations on youth health volunteers, many of whom serve without remuneration or adequate digital training. Even though young individuals are often referred to as “digital natives,” this idea should not become an alternative to proper training regarding digital literacy, ethical aspects, and proper usage of artificial intelligence-based technologies.

As machine learning models and automated decision tools continue to influence community-level health responses, youth volunteers may increasingly be required to implement system-recommended actions that are beyond their level of understanding and/or influence. There remain serious questions concerning implementation, ownership, and responsibility. Even more concerning are questions about privacy and consent when mobile devices collect private healthcare data transmitted over electronic platforms, especially when operating outside digital literacy. Unless these issues fall under an appropriate set of governance principles, youth volunteers could be at risk for problems related to data exploitation, public distrust, and system errors. In the absence of governance structures that explicitly support volunteer roles, digital health systems risk outsourcing institutional responsibilities to the unpaid labor of young people, while placing young volunteers at ethical, legal and professional risks beyond their skills and experience. Acknowledgment, oversight and accountability measures are therefore not merely desirable, but vital for effective digital health programs.

Nevertheless, the presence of health volunteers among the youth constitutes an important area that holds great potential to humanize and democratize digital health innovation. This is because youth health volunteers are well-positioned to identify the imperfections of the designs that exist in health technologies, including the need to be online at all times, language barriers that confuse the interface, and algorithms that do not understand the social determinants of health [9].

## Recommendations and future directions

The investment in a complete capacity-building program which is based on the knowledge of health, digital literacy, and ethics will give youth health volunteers the ability to become digital intermediaries. In this respect, the youth health volunteers will provide support to patients and navigate the increasingly complex digital health environment. From this perspective, this investment will play an integral part in youth development as the training provides the youth health volunteers with technology skills.

Supportive institutional contexts are also important. So digital health programs involving youth health volunteers will need to set expectations, provide mentoring and acknowledge that digital labor, which refers to the time and effort invested in using digital tools, entering data and communicating, is valuable to health systems. This is especially important when digital health research expands into greater levels of activity because a lack of recognition could lead to entrenching inequalities, especially if digital levels of activity increase. Youth-led networks and learning platforms are alternative building blocks for improving digital health engagement through volunteering [8].

The role of research organizations and funding bodies is, therefore, to ensure that youth health volunteers are incorporated in the production of digital health evidence, both as implementers and participants in research. Assessing the impact of artificial intelligence-driven tools and digital decision support systems should, therefore, evaluate the impact on youth health volunteers, among other factors. As digital health continues to expand, the importance of youth health volunteers in shaping equitable health provision will become increasingly significant.

## Conclusion

Considering youth health volunteers as partners, not as marginal users, will be key to ensuring that digital health systems designed in the future will be equitable, credible, and aligned with the needs of the population they serve. The centrality of youth voices in digital health governance, the empowerment of their capabilities, and their protection is fundamental to achieving an inclusive digital future for public health. Specifically, if these concerns remain unaddressed, the risk is that future digital health systems might become even less equitable, especially in low-resource areas. Digital health systems of the future must include youth volunteers not only as users but as strategic partners. In order to fulfill this vision, further research is needed on the impact of frameworks for the governance of AI, the provision of digital skills training, institutional support, and ethical considerations on the efficacy, sustainability, and engagement of youth health volunteers within varying health care systems. Such findings would be vital in the development of an inclusive digital health policy that ensures maximum benefit of artificial intelligence and protection of volunteer well-being and accountability.
